# *In situ* photo-crosslinked hydrogel promotes oral mucosal wound healing through sustained delivery of ginsenoside Rg1

**DOI:** 10.3389/fbioe.2023.1252574

**Published:** 2023-09-28

**Authors:** Jie Xu, Zhenghao Zhang, Xiaofeng Ren, Yunan Zhang, Yang Zhou, Xiaorong Lan, Ling Guo

**Affiliations:** ^1^ Department of Oral Prosthodontics, The Affiliated Stomatological Hospital of Southwest Medical University, Luzhou, China; ^2^ Institute of Stomatology, Southwest Medical University, Luzhou, China; ^3^ School of Stomatology, Southwest Medical University, Luzhou, China; ^4^ Oral and Maxillofacial Reconstruction and Regeneration of Luzhou Key Laboratory, Luzhou, China

**Keywords:** ginsenoside Rg1, hydrogel, visible-light crosslinking, oral mucosa wound, antiinflammation, tissue repair

## Abstract

Oral mucosal wounds exhibit an increased susceptibility to inflammation as a consequence of their direct exposure to a diverse range of microorganisms. This causes pain, slow healing, and other complications that interfere with patients’ daily activities like eating and speaking. Consequently, patients experience a significant decline in their overall quality of life. Therefore, the pursuit of novel treatment approaches is of great importance. In this study, ginsenoside Rg1, a natural active substance extracted from ginseng root, was chosen as a therapeutic agent. It was encapsulated in a screened photo-crosslinked hydrogel scaffold for the treatment of mucosal defects in the rat palate. The results demonstrated that Rg1-hydrogel possessed excellent physical and chemical properties, and that oral mucosa wounds treated with Rg1-hydrogel exhibited the greatest healing performance, as evidenced by more pronounced wound re-epithelialization, increased collagen deposition, and decreased inflammatory infiltration. Subsequent investigations in molecular biology confirmed that Rg1-hydrogel stimulated the secretion of repair-related factors and inhibited the secretion of inflammatory factors. This study demonstrated that the hydrogel containing ginsenoside Rg1 significantly promotes oral mucosal tissue healing *in vivo*. Based on the findings, it can be inferred that the Rg1-hydrogel has promising prospects for the therapeutic management of oral mucosal wounds.

## Graphical Abstract

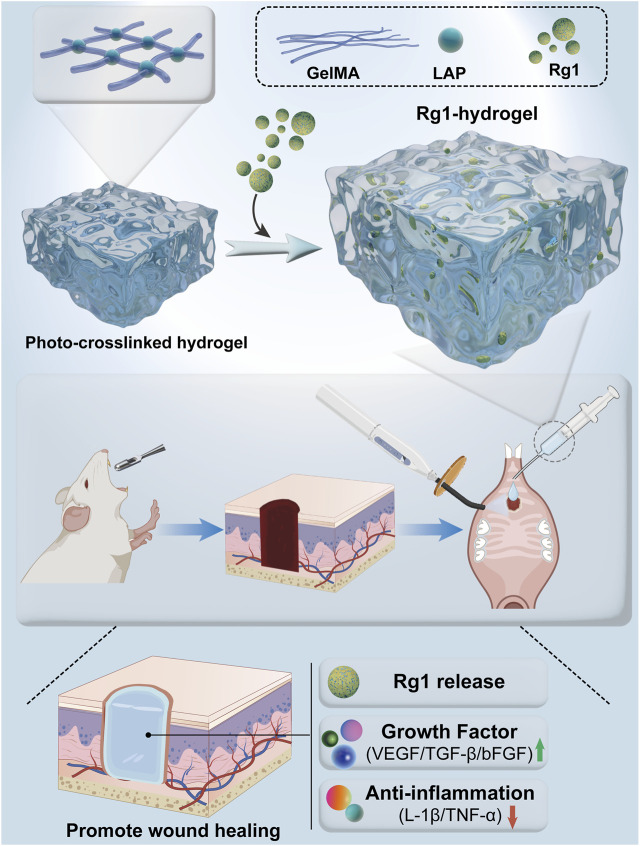

## 1 Introduction

The mucosa covers all oral surfaces except the teeth, serving as an inherent physiological barrier that protects against possible injuries encountered in everyday activities. It is also the first line of defense for our respiratory and digestive systems against various microorganisms (Suárez et al., 2021; Buggert, 2023). Oral mucosal wounds resulting from tooth extraction, oral surgery, or certain oral mucosal diseases are among the most prevalent oral soft tissue conditions manifesting as a loss of epithelial integrity (Toma et al., 2021). In addition, the pain and inflammation it causes can have a significant impact on patients’ daily activities, such as speaking and eating, and substantially reduce their quality of life (Xue et al., 2022; Salih et al., 2023).

The oral cavity remains moist as a result of saliva secretion, and muscle movement due to chewing and speaking also makes it show a highly dynamic characteristic (Zhang et al., 2021), these factors pose challenges in maintaining the therapeutic concentration of drugs locally. The types of agents commonly used clinically for treating such oral soft tissue diseases include mouthwashes, powders, sprays, etc., which are readily rinsed away by saliva or food, thereby diminishing their therapeutic effect (Suharyani et al., 2021). Therefore, there is an urgent need to develop drug-delivery materials that are better suited for topical treatment of oral mucosa.

Among various biomaterials, hydrogel materials have received extensive attention in the field of soft tissue wound healing due to their superior hydrophilicity, biocompatibility, and degradability (Chen et al., 2023; Shan and Wu, 2023; Solanki et al., 2023). In numerous studies, therapeutic components such as antibiotics (Ribeiro et al., 2022), glucocorticoids (Koh et al., 2023), stem cells (Qi et al., 2023), growth factors (Shan and Wu, 2023), etc. have been incorporated into different hydrogels to promote wound healing. However, chronic wounds caused by oral mucosal diseases may face challenges when using these drugs, which can have long-term side effects and are costly (D'Achille and Morroni, 2023; Hua et al., 2023). Accordingly, we propose to introduce a biomaterial that contains natural active ingredients, it can closely suit the affected area, resists fluid washing and oral movements, exerts an anti-inflammatory effect, and promotes tissue regeneration.

Ginseng is a natural herb with a variety of biological activities and has been used for two thousand years to treat different diseases (Potenza et al., 2023). Nowadays, ginseng is also widely used as an important nutritional supplement or medicinal ingredient worldwide (Chen et al., 2021). Ginsenoside Rg1 is a bioactive component extracted from ginseng, and its role in anti-oxidative stress and anti-apoptosis has been confirmed by numerous studies (Luo et al., 2020; Ren et al., 2021). In recent years, a growing number of studies have shown that it significantly promotes angiogenesis and remodeling (Chen et al., 2019; Shang et al., 2022), and hence ginsenoside Rg1 is expected to become a potential drug for the repair and treatment of oral soft tissue diseases. However, its clinical application is limited by its short half-life and absence of long-term retention in the injured area (Cheng et al., 2022; Yang et al., 2023). Therefore, it is a promising idea to discover an appropriate scaffold material that can slowly and continuously release Rg1 and remain stable at the wound site for a longer period of time to produce a therapeutic effect.

We selected injectable hydrogels that are photoinitiated cross-linked among a wide variety of hydrogels. On the one hand, it enables tight covering of the wound owing to its controlled sol-gel transformation process, which begins as a flowing liquid, in this process, it can actively adapt to any shape of tissue defects and quickly transform into a gel state following exposure to visible light of a certain wavelength (Nguyen and West, 2002). On the other hand, its injectable nature can also improve the convenience of treatment procedures (Salehi et al., 2023). Currently, gelatin methacryloyl (GelMA) is widely used in the field of tissue regeneration due to its excellent biocompatibility and physicochemical properties (Im and Lin, 2022; Kurian et al., 2022). Lithium phenyl-2,4,6-trimethylbenzoylphosphinate (LAP) is a commonly used visible light initiator. Studies have shown that LAP, as a photoinitiator of the hydrogel scaffold, has no effect on the cell viability of various cells encapsulated in hydrogels, exhibiting good biocompatibility (Lin et al., 2013; Kulkarni et al., 2022; Maiz-Fernández et al., 2022). The combination of GelMA and LAP can trigger the crosslinking process under the blue light activation of 405 nm wavelength and quickly form a gel state (Fairbanks et al., 2009). LAP enables the scaffold material to be activated by visible light rather than the extensively used ultraviolet light, thus avoiding a series of biosafety issues resulting from ultraviolet phototoxicity (Mullenders, 2018; Zhao et al., 2019). We speculate that ginsenoside Rg1-loaded photoinitiated cross-linked hydrogel may be a promising repair material. Prior to light curing, its sol form is highly mobile and can adapt to irregular oral soft tissue wounds. After 10 s of blue light irradiation, it transforms into a gel state, which is characterized by strong tissue adhesion and stability in the lesion area and plays a role in anti-inflammatory, angiogenesis, and tissue regeneration via the continuous release of Rg1.

Although hydrogels containing ginsenosides and other bioactive components have been observed to be effective in the treatment of bone tissue defects and periodontal inflammation (Guo et al., 2021; Wu et al., 2021), it is difficult to clarify whether the effect of promoting tissue repair is the result of ginsenosides or other active components because of their diverse therapeutic components. Our previous *in vitro* experiments have provided evidence of the anti-inflammatory and anti-apoptotic properties of ginsenoside Rg1, as well as its underlying biological mechanisms (Chu et al., 2023). In this work, we proposed preparing hydrogel scaffolds for oral mucosal wound repair by combining GelMA and LAP with a single bioactive component, ginsenoside Rg1. In a rat palatal mucosal defect model, the effect of Rg1-hydrogel on soft tissue repair was evaluated to assess the viability of topical application of ginsenoside Rg1 to promote soft tissue repair. It provides a theoretical foundation for the future clinical application of ginsenoside Rg1-loaded hydrogels in the treatment of oral mucosal diseases.

## 2 Materials and methods

### 2.1 Materials

Methacryloyl gelatin (GelMA, the amino substitution degree was 60%), Lithium phenyl-2,4,6-trimethylbenzoylphosphinate (LAP), and the portable curing light source facility (It emits visible light at 405 nm) were purchased from EFL-Tech Co., Ltd, Suzhou, China; Ginsenoside Rg1 was purchased from Beijing Solarbio Science & Technology Co., China; Human gingival fibroblasts (hGFs) were obtained from Oral & Maxillofacial Reconstruction and Regeneration of Key Laboratory, of which the identification has been completed in previous experiments of our research group (Huang et al., 2019). All media and reagents related to cell experiments were purchased from Thermo Fisher Scientific Inc., United States; The Cell Counting Kit-8 (CCK-8) assay kit was purchased from Dojindo Laboratories, Japan; The rats were obtained from the Experimental Animal Center of Southwest Medical University (Luzhou, China). All the reagents were of analytical grade and used as received.

### 2.2 Preparation of photocuring hydrogel

Photoinitiator LAP (0.05 g) was put into a dark brown glass bottle, added 20 mL of phosphate-buffered saline (PBS, 1×, pH 7.2–7.4, 0.01M; this specification of PBS was used in all subsequent experiments unless otherwise stated), and heated in a water bath at 40–50°C for 15 min, shocking it while heating. After this step is completed, the mixture is divided into two dark centrifuge tubes of 10 mL each; 0.6 g and 0.3 g of GelMA were placed, respectively, in centrifuge tubes containing 10 mL of the mixture. The mixture was heated in a water bath at 40–50°C for 30 min in the dark. During the period, it was repeatedly shaken to prepare a light-cured hydrogel precursor solution with GelMA concentrations of 3% w/v and 6% w/v, respectively. The solution was immediately filtered for subsequent experiments with a sterile needle filter (pore size of 0.22 μm). The precursor solution could be photo-polymerized under 405 nm visible light irradiation for 10 s to form the hydrogel.

### 2.3 Characterization of photocuring hydrogel

#### 2.3.1 Surface morphology observation

The morphology of the photocuring hydrogel was observed by scanning electron microscopy (SEM, Inspect F50, Thermo Fisher, United States). Hydrogels were prepared in 24-well plates using the method described above. Freeze them at −20°C for 2 h and then freeze-dry them in a vacuum freeze-dryer (LABCONCO, United States) for 18 h to prepare the sample. Samples were frozen in liquid nitrogen for 2 min before the test and then removed; they were cut open with a surgical blade to expose their cross sections. The cross-sections of all samples were sputter-coated with platinum for observation, and the cross-sectional morphology of the gel material was observed using three random images captured by SEM.

#### 2.3.2 Mechanical testing

The hydrogel precursor liquid was put into a mold (Φ10 × 10 mm), under irradiation with 405 nm visible light for 10 s, the gel was formed and then demoulded, and its mechanical properties were tested with a universal mechanical testing machine (INSTRON, 5965, Massachusetts, United States).

#### 2.3.3 Swelling behavior

The sample hydrogels were prepared in the mold (Φ20 × 20 mm) using the method described above and then freeze-dried as previously described, there were 3 replicates for each sample. The initial mass of the sample was recorded as M_0_, then the sample was immersed in phosphate-buffered saline (PBS) and placed in a constant temperature incubator (37°C). At different points in time, the immersed sample is taken out and carefully wiped off the surface moisture with filter paper, and the weight at this point was recorded as M_t_. The swelling ratio (SR) of the hydrogel was calculated according to the following equation:

$$SR\left(\%\right)=\frac{\left(M_{t}-M_{0}\right)}{M_{0}}\times 100$$

### 2.4 Preparation of Rg1-loaded photocuring hydrogel

The hydrogel material with better physicochemical properties was selected in previous experiments. Dissolve 20 mg of Rg1 in 2 mL of PBS solution to obtain a highly concentrated Rg1-PBS mixture for subsequent use. The drug-laden hydrogel precursor solution with Rg1 concentrations of 500 μg/mL and 1,000 μg/mL can be obtained by adding 50 μL and 100 μL of Rg1-PBS mixture to each 1 mL of hydrogel precursor solution using a high-precision micropipette, respectively. The Rg1-laden hydrogel precursor solution was converted into gel under 405 nm visible light irradiation, and we named them Rg1-500-Gel and Rg1-1000-Gel, respectively.

### 2.5 Characterization of chemical structure

The Fourier transform infrared spectroscopy (FTIR) analysis (Jasco, 6300, Tokyo, Japan) of Rg1 and the hydrogel sample was performed in the range of 400–4500 cm^−1^ to investigate any possible interaction between Rg1 and the raw material of the hydrogel (Song et al., 2020).

### 2.6 *In Vitro* Rg1 release

The release of Rg1 from Rg1-500-hydrogel and Rg1-1000-hydrogel was determined by high-performance liquid chromatography (HPLC). 1.5 mL of precursor solution of Rg1-500-hydrogel and Rg1-1000-hydrogel were placed into cylindrical molds with a diameter of 20 mm, respectively, and demoulded after light curing as described previously. The samples in each group were prepared for 3 replicates. The sample was placed in a 6-well plate, 5 mL of PBS solution was added to each well, and the plate was placed in a 37°C incubator, extracted 2 mL of the soaking solution every 24 h and supplemented with 2 mL of fresh PBS solution. The concentration of Rg1 in the extract was determined by HPLC (Agilent II 1260 Infinity LC, United States). The chromatographic column used in this experiment was Diamonsil C18 (4.6 × 250 mm, 5 μm), and the detection wavelength was 203 nm. (Xu et al., 2023).

### 2.7 *In Vitro* cytotoxicity evaluation

The cytotoxicity of the photocurable hydrogel with or without ginsenoside Rg1 towards human gingival fibroblasts (hGFs) was evaluated by the CCK-8 assay. The same size hydrogel (Φ10 × 10 mm) was immersed in 6 mL of complete growth medium (including 10% FBS, 1% penicillin-streptomycin solution, and basal DMEM medium) per well and placed in a cell incubator (37°C, 5% CO^2^). After 48 h, the hydrogel soaking solution was collected and filtered with a sterile needle filter (pore size 0.22 μm) for subsequent use. The hGFs were seeded into 96-well plates at a density of 1 × 105 cells/well and then treated with different soaking solutions. After 24, 48, and 72 h of incubation, the CCK-8 mixed medium was added to each well and measured at 450 nm on a microplate reader (SynergyH1, BioTek, United States).

### 2.8 *In Vivo* wound repair effect of Rg1-hydrogel

#### 2.8.1 Rat palatal mucosal defect model

Male Sprague Dawley (SD) rats (7–8 weeks) were used to create palatal mucosal defect models. The animal study was reviewed and approved by the Ethics Committee of Southwest Medical University (the approval number: 20230703-009).

SD rats were divided into 3 groups: the control, blank hydrogel, and Rg1-hydrogel groups. After being reared adaptively for 1 week, the rats were anesthetized via intraperitoneal injection of pentobarbital sodium (30 mg/kg body weight), and soft tissue defects were prepared in the middle of the rat’s palate with a gingival circumferential knife with an internal diameter of 2 mm, and the mucoperiosteal flap was removed to form a circular defect. The pre-hydrogel fluids with or without Rg1 were dropped onto the wound of the palate in the experimental groups with the Pasteur pipette, and the gel was formed under the irradiation of the visible light source, while the control group was not treated. The entire process of placing hydrogels on the rat oral mucosal wound is shown in Supplementary Movie S1. The hydrogels were changed every 24 h and pictures were taken at different time points to observe the healing of the wound over time. On days 1, 3, and 5, three rats in each group were euthanized by intraperitoneal injection of overdose pentobarbital sodium (80 mg/kg body weight), and the peri-wound tissues were taken and fixed in 4% paraformaldehyde solution at room temperature for subsequent analysis.

#### 2.8.2 Wound healing analysis

The recovery of the wound area in the rat’s mouth was recorded photographically with a high-definition camera. HE and Masson staining were used for histological analysis of the collected tissues, and the pictures of the slices were captured by a slide scanning system (Leica, Wetzlar, Germany). The tissue regeneration and inflammation around the wound were detected by immunofluorescence staining. Pictures were taken with a fluorescence microscope (BX53, Olympus, Tokyo, Japan). Real-time quantitative polymerase chain reaction (RT-qPCR) and Western blot experiments were used to analyze the expression of inflammatory factors and repair-related factors in the soft tissue around the wound. The primer sequences were researched and presented in the (Supplementary Table S1), and the results were standardized against the CT value of Glyceraldehyde-3-phosphate dehydrogenase (GAPDH) and analyzed using the 2^−ΔΔCT^ method.

### 2.9 Statistical analysis

All data were presented as the mean ± standard deviation. Comparisons between groups were performed by applying one-way ANOVA under Tukey’s test using GraphPad Prism (GraphPad Software, United States). Differences were considered statistically significant at *p < 0.05, **p < 0.01, ***p < 0.001, ****p < 0.0001.

## 3 Results and discussion

### 3.1 Synthesis and characterization of photocurable hydrogels

**FIGURE 1:**
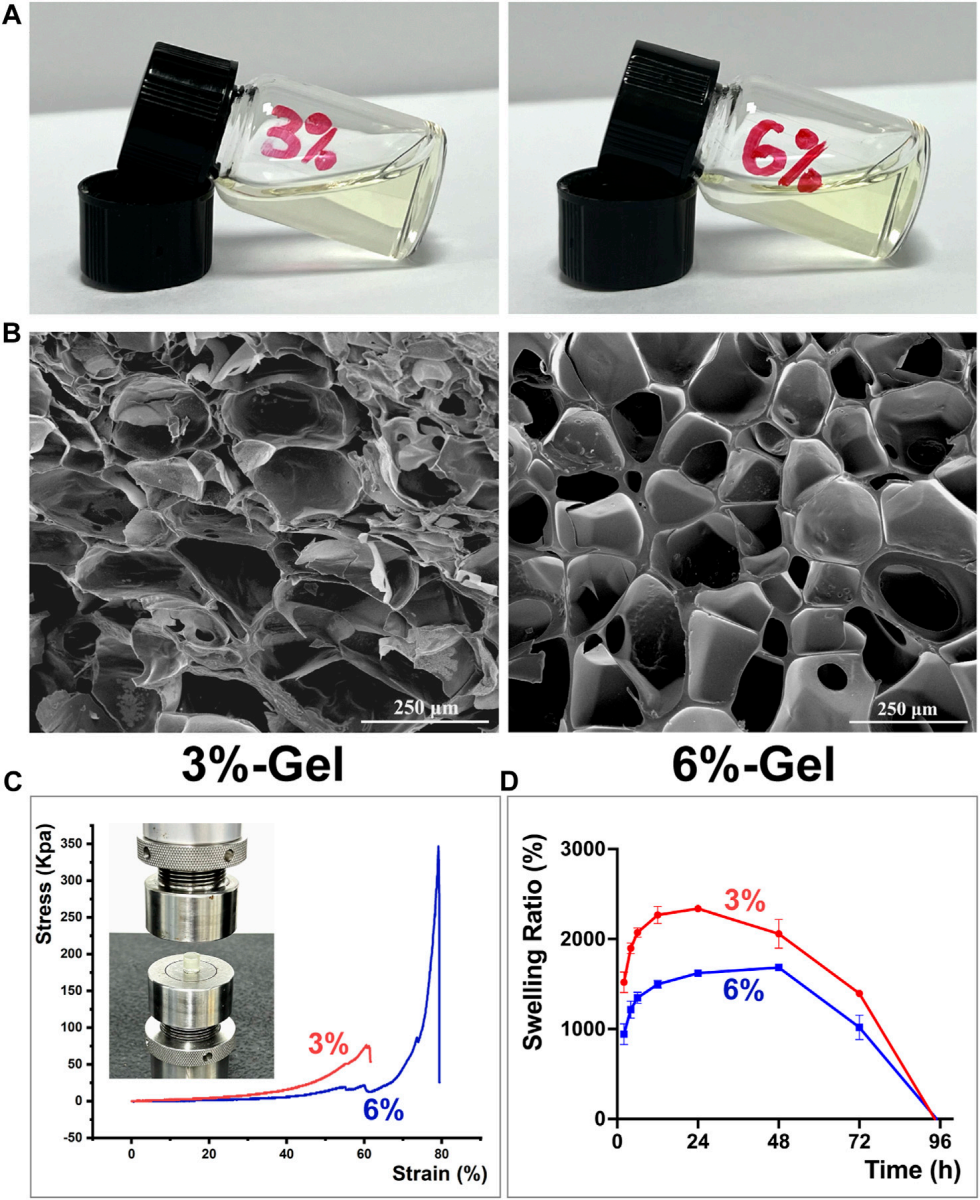
**(A)** Representative formation of the hydrogels in a glass bottle. **(B)** SEM images. **(C)** The compressive stress of hydrogels. **(D)** Swelling study of Hydrogels. The error bar indicates the standard deviation (*n* = 3).

At present, there exists an abundance of hydrogel materials that are utilized in the field of wound healing. Some of them promote tissue repair through the hydrogel material components themselves, such as chitosan (Hamedi et al., 2018), hyaluronic acid (Graça et al., 2020), silk fibroin (Gholipourmalekabadi et al., 2020), etc., while others reduce peri-wound inflammation and promote tissue repair by loading biologically active ingredients, such as antibiotics, nanoparticles, stem cells, and growth factors (Ferreira et al., 2018; Fan et al., 2019; Liang et al., 2022). The previous study conducted by our research group demonstrated that ginsenoside Rg1 exhibits notable antioxidant and anti-apoptotic properties (Chu et al., 2023). Furthermore, it has been confirmed that Rg1 plays a distinct function in facilitating vascular regeneration (Chen et al., 2019). Based on the above information, it may be inferred that Rg1 has significant potential for application in the field of tissue regeneration. However, the majority of these investigations are focused on *in vitro* experiments. At the moment, we intend to use a single bioactive component, ginsenoside Rg1, piggybacked on a basic hydrogel scaffold for the restorative treatment of oral mucosal defects in rats, in order to preliminarily explore the potential application of Rg1 in the field of tissue regeneration. Excellent physical and mechanical properties are critical to the selection of hydrogel scaffold materials for carrying therapeutic components (Atia et al., 2023). In order to screen out better hydrogel scaffolds carrying the target drug ginsenoside Rg1, we evaluated the performance of hydrogel scaffolds with GelMA concentrations of 3% w/v and 6% w/v, respectively. We synthesized the 3% and 6% of the scaffold materials (hereinafter referred to as 3%-Gel and 6%-Gel) as shown in Figure 1A and observed their microscopic morphology using scanning electron microscopy, which showed that both concentrations of scaffold materials exhibited an interconnected pore structure. As shown in Figure 1B, for the porous structure of 3%-Gel, the pore wall is thin and easily broken, and many pore wall fragments can be seen in the electron microscope image. For 6%-Gel, the pore wall was thick and regular, and no debris was found. The ImageJ (ImageJ 2.3.0 software, United States) was used to analyze three random images collected by SEM. It was found that the pore diameter of 3%-Gel was about 0–282.9 μm, and that of 6%-Gel was about 0–200.7 μm. With the concentration of GelMA increasing from 3% to 6%, the average pore diameter decreased from 196.3 ± 42.9 μm to 164.8 ± 33.3 μm (Supplementary Figure S1). The 6%-Gel scaffold is expected to withstand a greater biomechanical load and provide physical space for the growth of cells from the surrounding tissues, as shown by the compression curve in Figure 1C, where the compressive strength of the 6%-Gel scaffold is significantly higher than that of the 3%-Gel scaffold (He et al., 2000).

Superior swelling property is a crucial factor for assessing the application potential of hydrogel scaffold materials, as stable swelling performance can prolong the release of encapsulated pharmaceuticals (Wang et al., 2020). As demonstrated in Figure 1D, the 3%-Gel has a higher swelling rate than the 6%-Gel, but it reaches its maximum mass between 24 and 48 h and the mass reduction occurs before 48 h, whereas the mass of the 6%-gel begins to decline after 48 h, showing a more stable swelling performance. The freeze-dried hydrogel scaffold is shown in Supplementary Figure S2A. During 24 h of immersion in PBS at 37°C, the diameters of both 3%-Gel and 6%-Gel increased due to water absorption. However, compared with 3%-Gel, the shape of 6%-Gel is more regular and stable (Supplementary Figures S2B, C). After 24 h, 3%-Gel begins to degrade. Overall, the 6%-Gel displayed an excellent balance between its physical properties and swelling behavior. Therefore, all of the following experiments were conducted with 6%-Gel.

### 3.2 Synthesis and characterization of hydrogels loaded with ginsenoside Rg1

**FIGURE 2:**
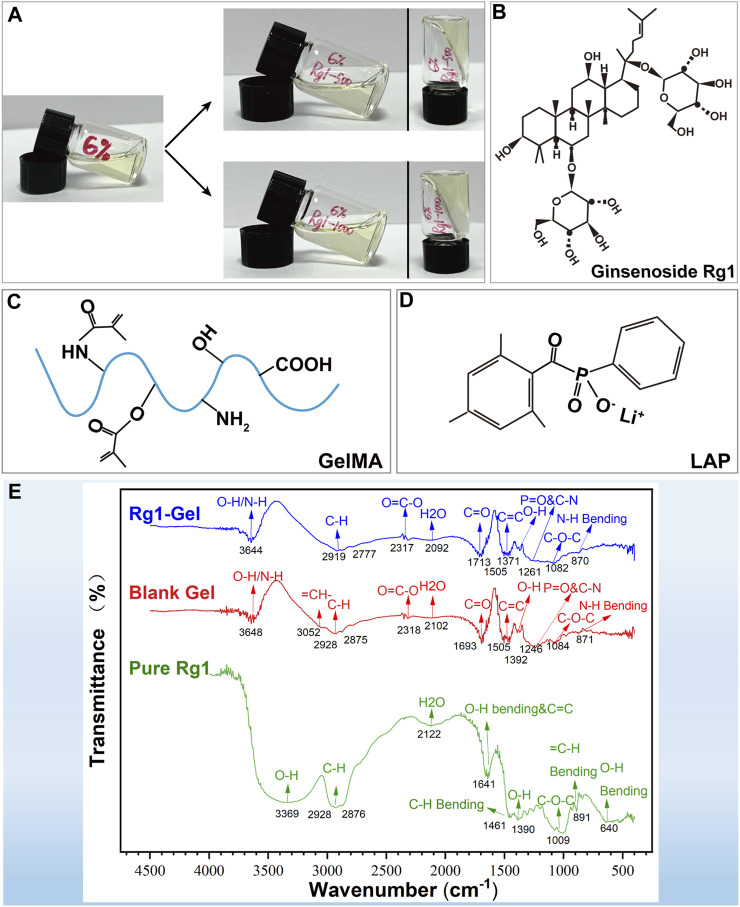
**(A)** Representative formation of the Rg1-loaded hydrogels in a glass bottle. **(B)** The molecular structure diagram of ginsenoside Rg1. **(C)** The molecular structure diagram of GelMA. **(D)** The molecular structure diagram of LAP. **(E)** FTIR spectra of pure Rg1, Blank Gel, and Rg1-Gel.

The hydrogel scaffold with GelMA concentrations of 6% was selected to carry ginsenoside Rg1 with concentrations of 500 μg/mL and 1000 μg/mL (hereinafter referred to as Rg1-500-Gel and Rg1-1000-Gel), respectively (Figure 2A). In order to further explore the chemical structure and interaction between ginsenoside Rg1 and hydrogel scaffold materials, the pure extract of ginsenoside Rg1, blank hydrogel, and Rg1 loaded hydrogel were measured and compared by the Fourier transform infrared spectroscopy (FTIR). The molecular structure diagram of ginsenoside Rg1 and the raw materials of hydrogel scaffolds (GelMA and LAP) are shown in Figures 2B–D. As demonstrated in Figure 2E, the FTIR spectra of ginsenoside Rg1 exhibited characteristic absorption peaks at 3,369 cm^−1^ and 1390 cm^−1^ (trough position), corresponding to the O-H stretching, which may be related to the high O-H content of Rg1 (Salarian et al., 2016). In the spectra of Blank Gel, characteristic peaks appeared near 1700 cm^−1^ and 871 cm^−1^, corresponding to the characteristic functional groups C=O and N-H of GelMA, and the peaks located in the range of 1454 cm^−1^ to 1505cm^-1^ were attributed to the characteristic structure of the benzene ring and functional group P=O (1261cm^−1^) of the photoinitiator LAP (Xu et al., 2022; Zhou et al., 2023). After loading ginsenoside Rg1 into the hydrogel scaffold, the spectra of Rg1-Gel revealed that the addition of Rg1 had no effect on the overall structure of Blank Gel. Possibly due to the concentration of Rg1 being relatively low, the absorption of Rg1 was obscured by the absorption of hydrogel scaffold functional groups, resulting in the variation trend of the Rg1-Gel spectrum exhibiting essentially the same characteristics as those of Blank Gel, with the exception of a slight increase in absorption intensity. Therefore, it can be concluded that the incorporation of Rg1 into the hydrogel did not alter its fundamental structure.

### 3.3 *In Vitro* Rg1 release and *in vitro* biocompatibility

**FIGURE 3:**
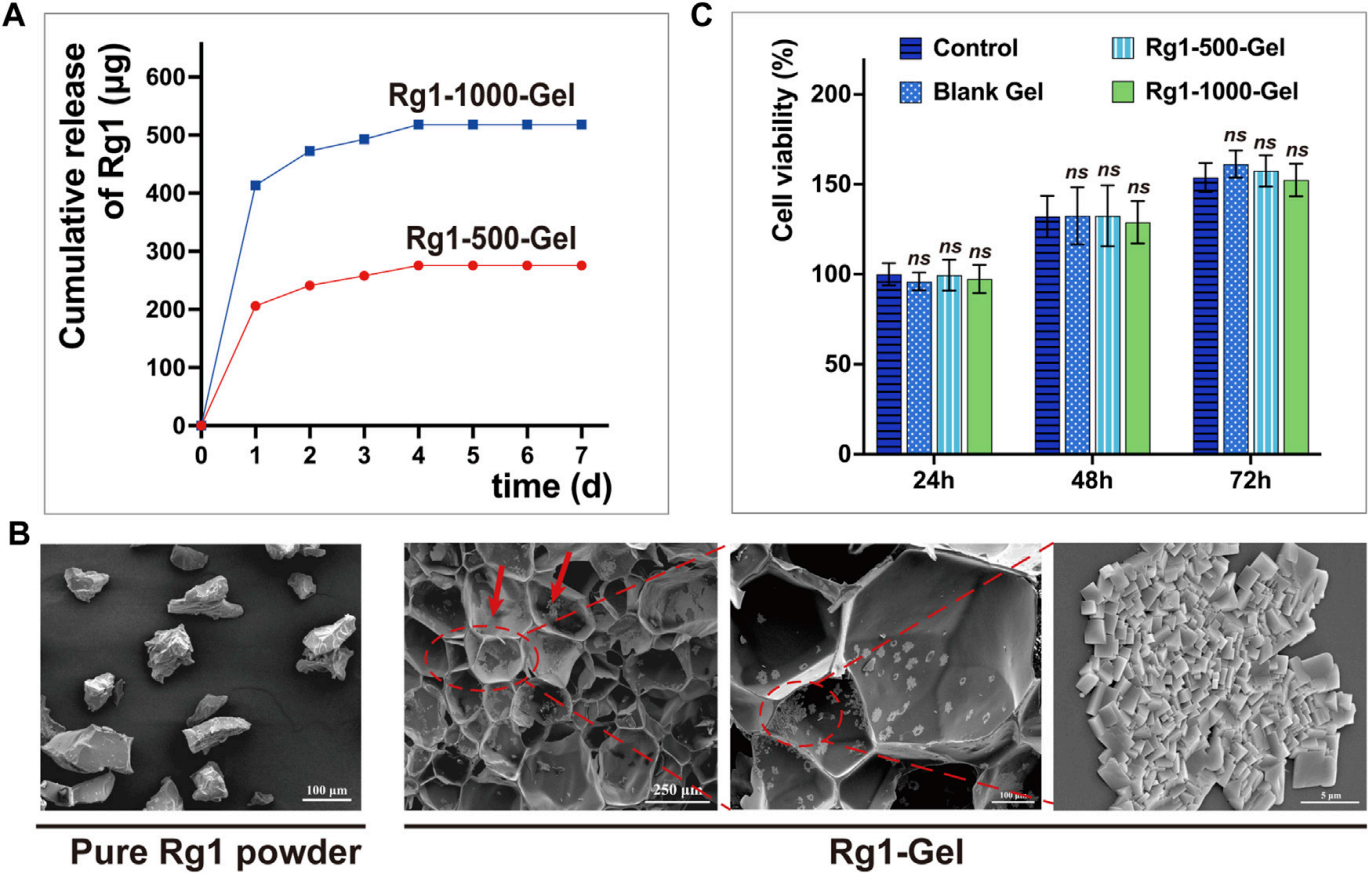
**(A)** The amount of Rg1 released from hydrogels which is measured by High-Performance Liquid Chromatography (HPLC). **(B)** SEM images. The red arrow indicates the Rg1 particles attached to the pore wall. **(C)** The cytocompatibility of the Rg1-Gels against human gingival fibroblasts (n = 5).

To determine whether Rg1-loaded hydrogels can deliver the bioactive drug Rg1 in a sustained and efficient manner, we analyzed the Rg1 concentration in the soaking solution of the hydrogels using high-performance liquid chromatography (HPLC). In our 7-day drug release assay, hydrogels loaded with two different concentrations of Rg1 showed a sustained release of Rg1 for more than 4 days, as illustrated in Figure 3A. Based on the outcomes of our group’s prior *in vitro* investigations concerning ginsenoside Rg1, we selected the hydrogel containing Rg1 at a concentration of 1,000 μg/mL (Rg1-1000-Gel) for the follow-up experiments. This particular formulation was chosen due to its ability to release a drug concentration roughly comparable to the previously determined optimum biological concentration of Rg1 (Chu et al., 2023). Under a scanning electron microscope (SEM), we observed Rg1-1000-Gel and determined that Rg1 had no significant effect on the porous structure of the hydrogel scaffold; however, particle deposition was observed on the pore wall. Compared to pure Rg1 powder, its morphology was found to be consistent (Figure 3B). We therefore speculated that the particles attached to the pore wall could be a part of the ginsenoside Rg1 loaded onto the hydrogel scaffold, while the other part of the Rg1 was encapsulated inside the hydrogel material. This inference also corresponds to the rapid release of Rg1 on the first day and slow release on the following days in the release experiments of Rg1 (Figure 3A). Excellent cytocompatibility is an essential requirement for the *in vivo* use of biomaterials (Oladipo et al., 2023). The Figure 3C shows the cell viability of gingival fibroblasts treated with the soaking solution of blank hydrogels and hydrogels loaded with different concentrations of Rg1 (Rg1-500-Gel and Rg1-1000-Gel). At 24, 48, and 72 h, there was no significant difference in cell viability (*p* > 0.05) between the hydrogel scaffold groups with or without Rg1. This result suggests that both the hydrogel scaffold and the Rg1-loaded hydrogel have good biocompatibility, providing a credible premise for subsequent *in vivo* experiments.

### 3.4 *In Vivo* wound healing studies

#### 3.4.1 Observation of the wound healing process

**FIGURE 4:**
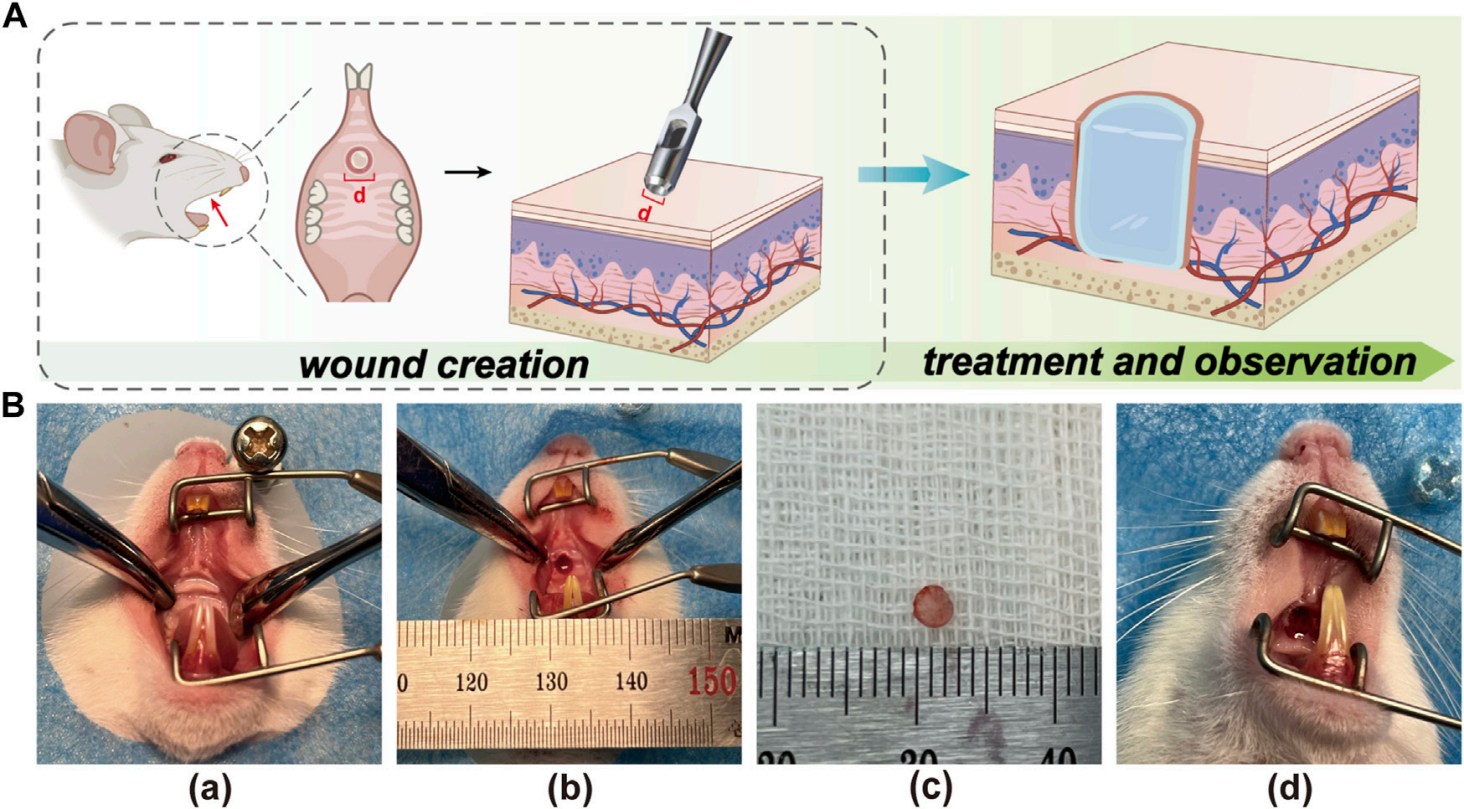
**(A)** The schematic illustration of creating the rat palatal mucosal defect model (defects with a diameter of 2 mm). **(B)** Recorded photographs of palatal mucosal wound creation and hydrogel placement in rats. **(a)**. Before wound creation. **(b)**. Wound creation. **(c)**. Mucosal tissue removed from the wound site. **(d)**. After hydrogel placement on the wound.

It is well known that positive results from *in vivo* experiments are a prerequisite for determining whether therapeutic biomaterials have the potential for clinical application (Farram et al., 1978). Previous *in vitro* experiments evaluated the feasibility of Rg1-loaded hydrogels for animal testing. To confirm the exact pro-wound healing effect of Rg1-Gel, we conducted *in vivo* experiments. In order to test the therapeutic effect of Rg1-Gel for oral mucosa defect treatment, we used the hydrogels with or without Rg1 (Rg1-Gel group and Blank Gel group) to repair the 2-mm diameter palatal mucosa defect in rats, all of which were deep enough to reach the bony surface of the palate, while the control group was not treated. The schematic diagram of animal experiment is shown in Figure 4A. The Figure 4B (a-d) shows the process of establishing the model and treating it with hydrogels.

**FIGURE 5:**
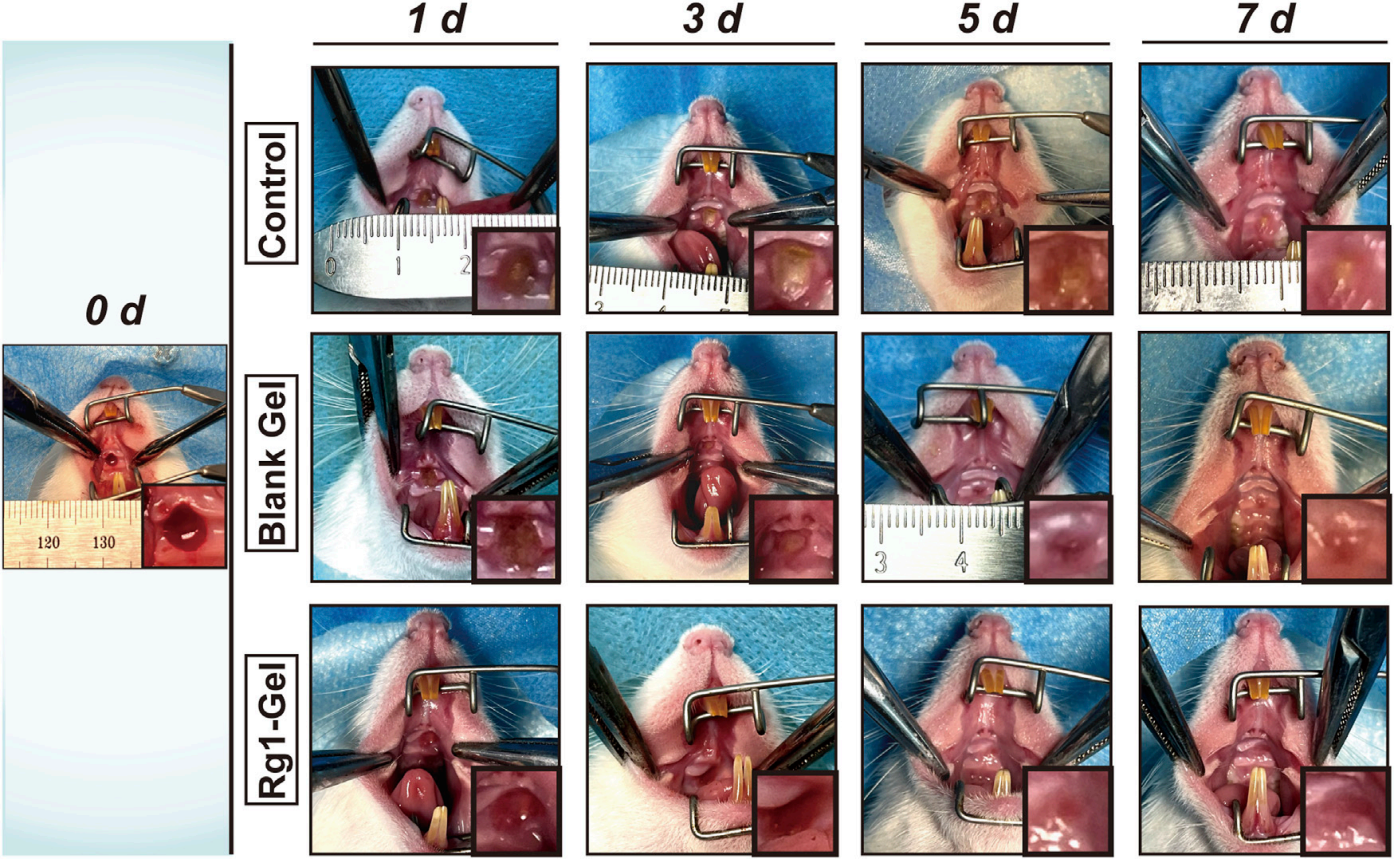
Photographs of wounds of the control group, Blank Gel group, and Rg1-Gel group on days 0, 1, 3, 5, and 7.

Subsequently, we monitored and recorded the entire healing process of the wounds and compared the healing effects of each group at specific time points. As can be seen from Figure 5, better healing was observed in the Rg1-Gel group at all time points. To be specific, on days 1, 3, and 5, distinct yellow-brown inflammatory exudate was observed on the mucosal wound surface in the control group; a smaller amount of exudate was observed in the Blank Gel group; and almost no inflammatory exudate was seen in the Rg1-Gel group. In addition, on the last day of our observation period (day 7), we found that although traces of trauma were still observed in the palatal mucosa of the Rg1-Gel group, the wound surface was completely covered by neonatal epithelial tissue. In the Blank Gel group, the wound marks were more visible, and there was a small area of incomplete healing in the center of the wound; however, in the control group, this incomplete healing was more severe, and a very small amount of yellow-brown exudate was observed in the wound center (Figure 5).

#### 3.4.2 Histological evaluation

**FIGURE 6:**
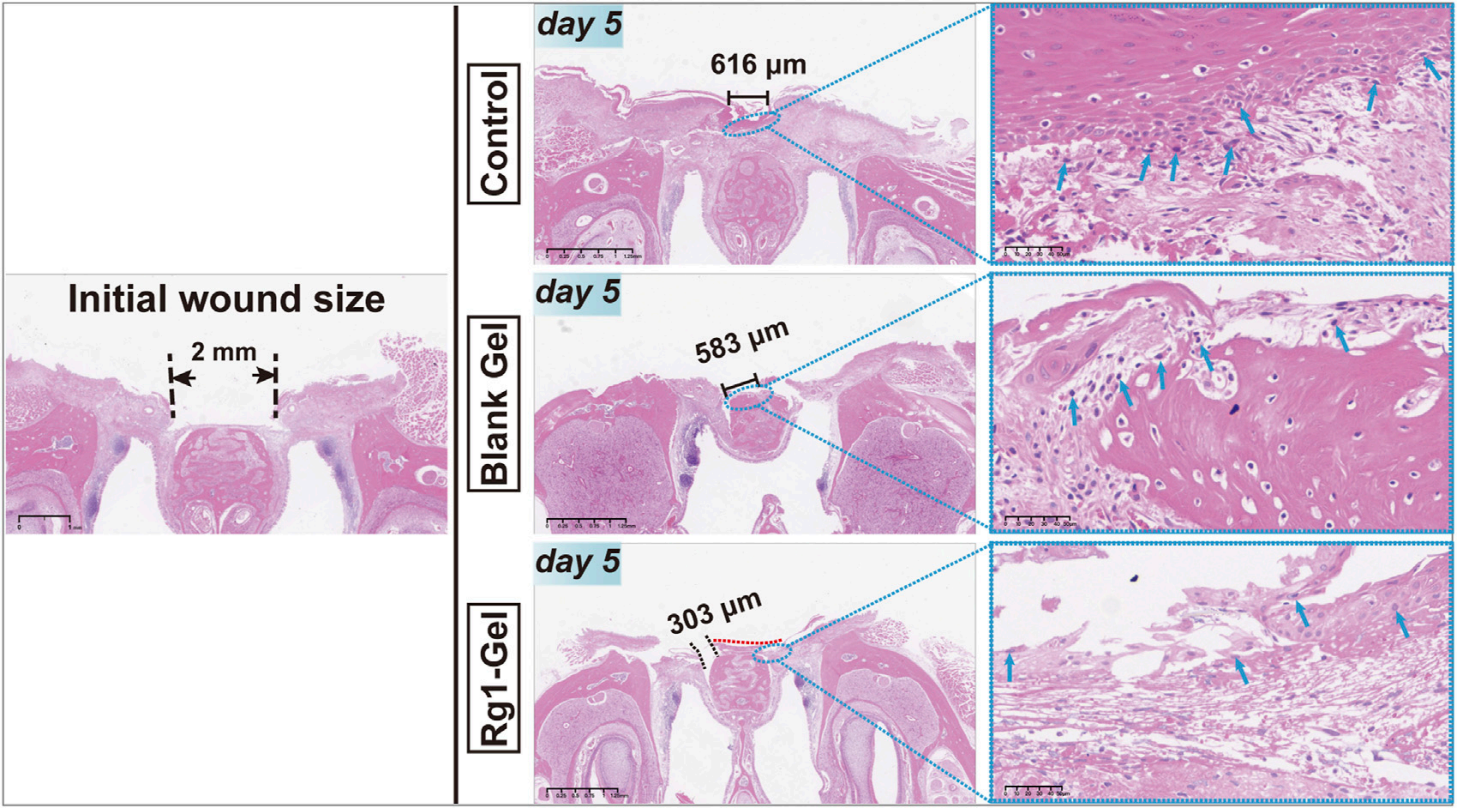
H&E staining of rat palatal mucosal defect for the Control, Blank Gel, Rg1-Gel groups on day 5.

In order to further evaluate the wound healing process, HE staining was performed on the peri-incisional tissues collected on day 5 (day 5 was selected because we observed the entire healing process and found that mucosal wounds healed most obviously but were not completely healed on the 5th day, allowing us to compare the healing effect among the different groups). Due to the diversity of oral microorganisms, oral mucosal wounds are more likely to cause inflammation and delayed healing than wounds on the skin (Costa et al., 2014; Menezes et al., 2022). As shown in Figure 6, more inflammatory cells (shown by the blue arrow) could be observed in the control group; the Blank Gel group showed a slight decrease in inflammatory cells compared with the control group, while the Rg1-Gel group showed the least distribution of inflammatory cells. Although the three groups showed varying degrees of healing, it was clear that the Rg1-Gel group exhibited the best healing performance, and significant neoepithelial tissue growth was observed (under the red dotted line). The largest unhealed area was observed in the control group (black solid line), and we speculate that this may be related to the previously observed inflammation.

**FIGURE 7:**
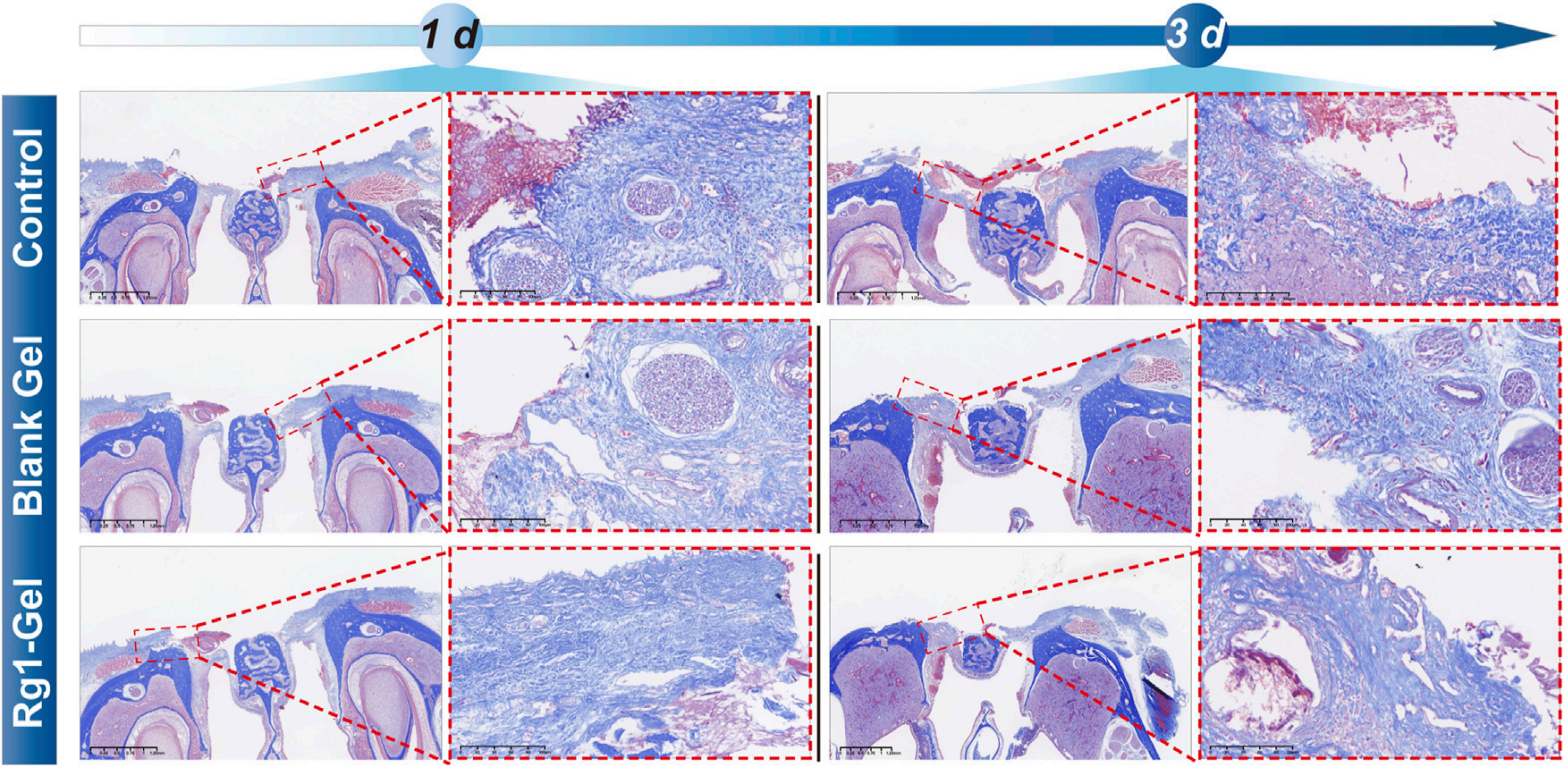
Masson staining of rat palatal mucosal defect for the Control, Blank Gel, Rg1-Gel groups on day 1 and day 3.

In the early stage of epidermal and mucosal wound healing, the synthesis of various types of collagen increases, replacing necrotic tissue and promoting healing (Gajbhiye and Wairkar, 2022). Hence, the deposition of collagen fibers serves as a crucial signal for assessing the process of wound healing. Masson staining was used to examine the deposition of collagen inside the tissue surrounding the incision. The Masson staining results presented in Figure 7 revealed that on day 3, the tissues surrounding the wound in the control group showed a small amount of collagen fiber deposition with a loose and disorganized fiber distribution, whereas the Blank Gel group displayed a slight increase in collagen fiber deposition, but its distribution remained loose and disorganized. In contrast, the orderly accumulation of collagen fibers in the Rg1-Gel group suggests that the treatment promotes wound healing. In conclusion, the histological analysis revealed that Rg1-Gel significantly accelerated the wound healing process.

#### 3.4.3 Assessment at the cellular or molecular level

##### 3.4.3.1 Rg1-Gel promotes regeneration of oral mucosa

**FIGURE 8:**
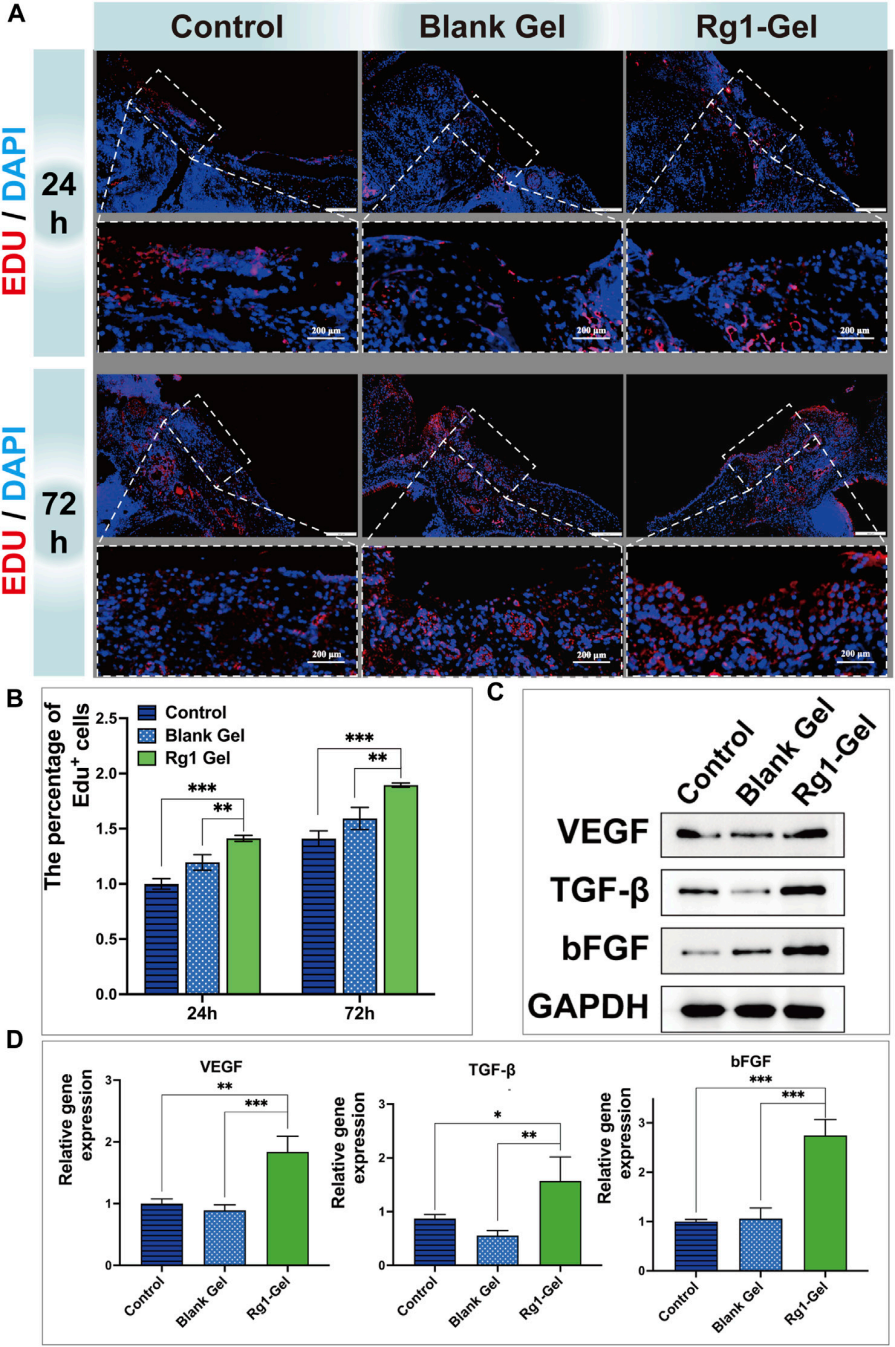
**(A)**. Edu-stained proliferative cells in the defect region at 24 and 72 h after operation **(B)**. Percentage of Edu-positive cells in the defective region at different time points **(C)**. Cytokine protein expression associated with soft tissue repair **(D)**. expression of soft tissue repair-related genes.

Immunofluorescence staining was performed on the tissues surrounding the wound to further investigate how Rg1-Gel promotes soft tissue repair at the cellular level (Zhang et al., 2021). EdU (5-Ethynyl-2′-deoxyuridine) is a thymidine analogue that can penetrate into the replicating DNA molecules instead of thymine (T) during cell proliferation and then quickly label newborn cells through a specific reaction based on EdU and fluorescent dyes (Fujita et al., 2022; Beauchemin et al., 2023). As shown in Figure 8A, more new proliferating cells (EDU-positive, EDU^+^) were observed in the tissues around the wound in the Rg1-Gel group at both time points, and the percentage of EDU-positive cells is shown in Figure 8B. This indicates that active cell proliferation is induced in response to the treatment of Rg1-Gel. Since the wound healing process is strictly regulated by various cytokines (Werner and Grose, 2003), we analyzed the expression of certain factors associated with soft tissue repair in peri-wound tissue using RT-qPCR and Western blot experiments.

Angiogenesis has been recognized as a crucial mechanism in the process of wound healing (Han et al., 2022). Vascular Endothelial Growth Factor (VEGF) has been considered one of the most potent angiogenic growth factors to initiate angiogenesis for a long period of time (Ong and Dilley, 2018). Numerous studies have shown that the basic fibroblast growth factor (bFGF) and transforming growth factor-beta (TGF-β) are key regulators of the soft tissue repair process (Wang and Li, 2023). Consequently, in this study, VEGF, bFGF, and TGF-β were selected as indicators to evaluate the effect of Rg1-Gel on the repair of oral mucosal tissue. As shown in Figure 8C and Supplementary Figure S3A, the VEGF protein detected by Western blotting was highly expressed in the wounds of the Rg1-Gel group (*p* < 0.001), and the protein levels of cytokines bFGF and TGF-β were also significantly increased in the Rg1-Gel group compared with the control group (*p* < 0.0001). Meanwhile, the gene expression of VEGF, bFGF, and TGF-β measured by RT-qPCR showed consistent results with the Western blot analysis (Figure 8D).

##### 3.4.3.2 Rg1-Gel regulates the inflammatory response in oral mucosal wounds

**FIGURE 9:**
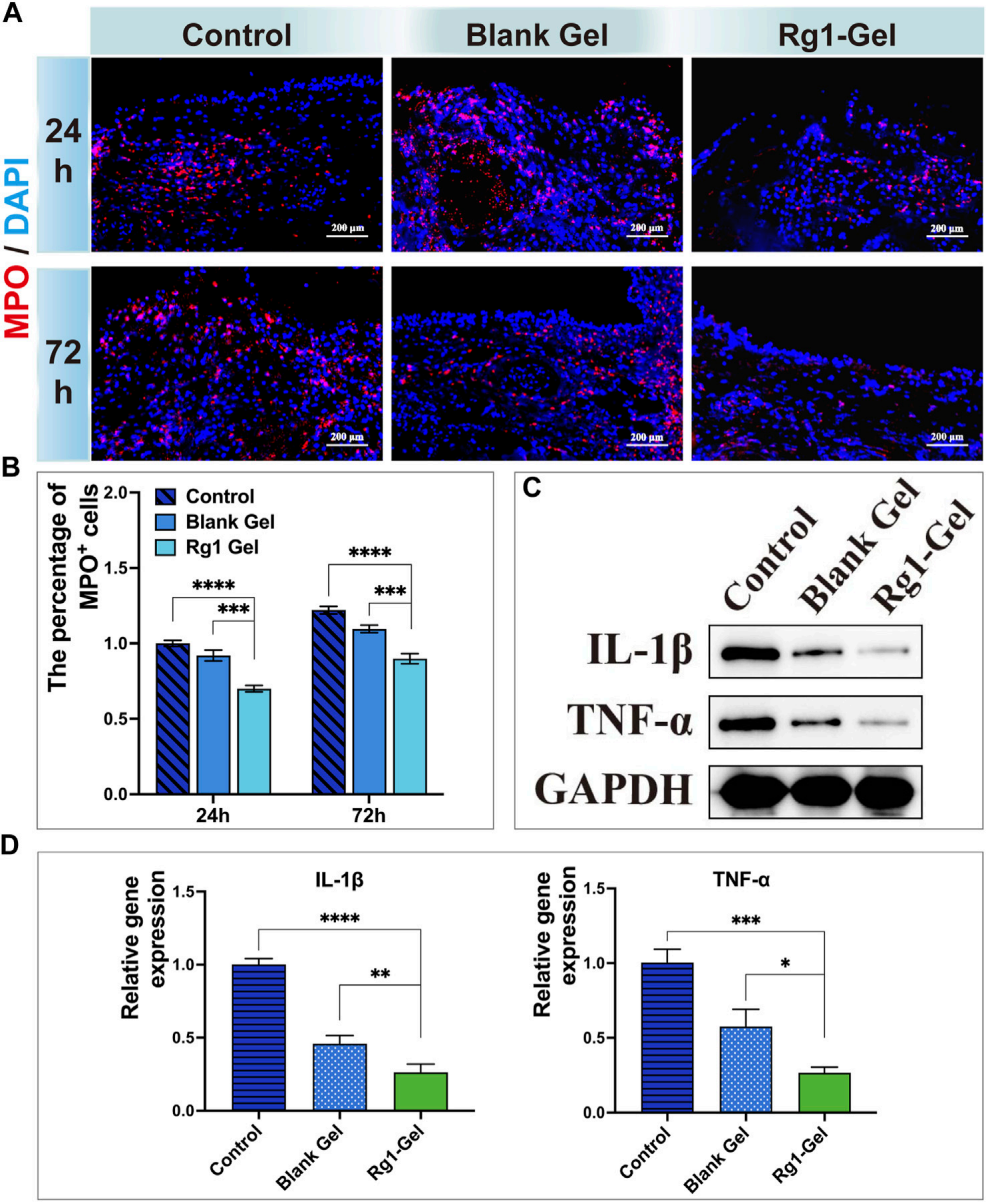
**(A)**. MPO-stained neutrophils in the defect region at 24 and 72 h after operation. **(B)**. Percentage of MPO-positive neutrophils in the defective region at different time points. **(C)**. Inflammation-associated cytokines protein expression. **(D)**. Expression of inflammation-associated genes.

Wound healing is a precisely regulated process consisting of four phases: hemostasis, inflammation, proliferation, and remodeling (Cai et al., 2023), of which the early inflammatory phase is dominated by the recruitment of neutrophils. Numerous studies have shown that effective inflammation inhibition can promote wound healing (Reinke and Sorg, 2012). Therefore, we chose to monitor the activity of neutrophils to evaluate the inflammatory response during wound healing. Myeloperoxidase (MPO) is a functional marker and activation marker of neutrophils, and its level and activity represent the function and activity of polymorphonuclear neutrophils (PMN). A large number of studies have shown that MPO can be used as a marker to indicate the inflammatory infiltration of tissues (Roumeguère et al., 2023). As shown in Figure 9A, at both time points, the Rg1-Gel group observed the fewest inflammatory cells (MPO-positive, MPO^+^) in the tissue surrounding the wound, and the percentage of MPO-positive cells is shown in Figure 9B. This suggests that Rg1-Gel treatment potentially has an anti-inflammatory effect on wound healing. In addition, the protein expression levels of inflammation-related factors (TNF-α and IL-1β) were the lowest in the Rg1-Gel group (*p* < 0.0001, Figure 9C, S3B), which was consistent with the results of its gene expression (Figure 9D). Since Rg1 showed good anti-inflammatory effects in the previous studies of our group (Chu et al., 2023) as well as *in vitro* studies by other scholars (Alolga et al., 2020), combined with the *in vivo* experimental results in this study, we speculate that the anti-inflammatory properties of Rg1-Gel can be attributed to the sustained release of Rg1.

## 4 Conclusion

In the present study, photoinitiated cross-linked hydrogel scaffolds containing ginsenoside Rg1 were utilized to treat oral mucosal wounds. These hydrogels exhibit an ordered porous morphology, excellent biocompatibility, and sustained Rg1 release properties. Our *in vitro* and *in vivo* investigations demonstrated that wounds treated with hydrogels containing ginsenoside Rg1 exhibited better wound contraction, more significant wound re-epithelialization and collagen deposition, more secretion of soft tissue repair-related factors, and less secretion of inflammatory factors, thereby promoting the healing of oral mucosal wounds, which was attributed to the sustained release of Rg1.

Nowadays, a variety of biological materials for tissue repair have emerged in an endless stream, and the active substances carried by biological materials are also diverse. We utilized a basic scaffold material to deliver a single bioactive substance Rg1, and confirmed its efficacy through *in vitro* and *in vivo* experiments, indicating that ginsenoside Rg1 will be a promising bioactive agent for the repair of oral soft tissue wounds. It is anticipated that this will provide a theoretical foundation for a broader application of Rg1 in biomaterials in the future.

## Data Availability

The original contributions presented in the study are included in the article/Supplementary Material, further inquiries can be directed to the corresponding author.
