# Supplementary material for: In situ photo-crosslinked hydrogel promotes oral mucosal wound healing through sustained delivery of ginsenoside Rg1
Source: Front Bioeng Biotechnol. 2023 Sep 28;11:1252574. doi: 10.3389/fbioe.2023.1252574 (PMC10569426; doi:10.3389/fbioe.2023.1252574)
Supplement: Supplementary file 1 [file DataSheet1.PDF]

frontiers

*Supplementary Material*

# In situ photo-crosslinked hydrogel promotes oral mucosal wound healing through sustained delivery of ginsenoside Rg1

**Jie Xu, Zhenghao Zhang, Xiaofeng Ren, Yunan Zhang, Yang Zhou, Xiaorong Lan, Ling Guo***

*** Correspondence:** Ling Guo (gl2005202@foxmail.com)

## Material and methods

### Animals

In this experiment, male SD rats (7-8 weeks) were used to create palatal mucosal defect models. Healthy male rats of similar body weight (200-220 g) were obtained from the Experimental Animal Center of Southwest Medical University (Luzhou, China) and were housed in an SPF-level environment with free access to food and water. Animals were allowed to acclimate for at least 7 days before the experiments. All animal experiments included in this study were approved by the Ethics Committee of Southwest Medical University (approval number: 20230703-009).

### Anesthesia & Euthanasia

In this experiment, anesthesia (30 mg/kg body weight) was performed by intraperitoneal injection of 2% pentobarbital sodium ($C_{11}H_{17}N_2NaO_3$, 2 g of pentobarbital sodium dissolved in 100 ml of physiological saline), and after the surgery, they were kept warm with a thermostatic blanket

until they woke up. At each time point, rats were sacrificed by intraperitoneal injection of an overdose of 2% pentobarbital sodium (80 mg/kg body weight) to collect samples. All procedures in this experiment were carried out in accordance with the *Guide for the Care and Use of Laboratory Animals*.

## Supporting information in Material and methods

***Supplementary Movie 1.*** **Oral mucosal wound healing assay by Rg1-Gel application in vivo on rat palatal mucosal defect model.** The video shows the whole process of placing Rg1-Gel on the oral mucosal wound of rats.

***Table S1.*** Primer sequences used in this study

| Gene name | Primer Sequences | fragment size (bp) |
|---|---|---|
| VEGF-F | CAGCTATTGCCGTCCAATTGA | 131 |
| VEGF-R | CCAGGGCTTCATCATTGCA | |
| TGF-β-F | ACCGCAACAACGCAATCTAT | 206 |
| TGF-β-R | ACCAAGGTAACGCCAGGAAT | |
| bFGF-F | ATCAAGGGAGTGTGTGCG | 173 |
| bFGF-R | CCAGTTCGTTTCAGTGCC | |
| IL-1β-F | CAAGCAACGACAAAATCCC | 147 |
| IL-1β-R | CAAACCGCTTTTCCATCTTC | |
| TNF-α-F | CCACGCTCTTCTGTCTACTG | 145 |
| TNF-α-R | GCTACGGGCTTGTCACTC | |

| | | |
|---|---|---|
| GAPDH-F | CAAGTTCAACGGCACAG | 138 |
| GAPDH-R | CCAGTAGACTCCACGACAT | |

## Results and discussion

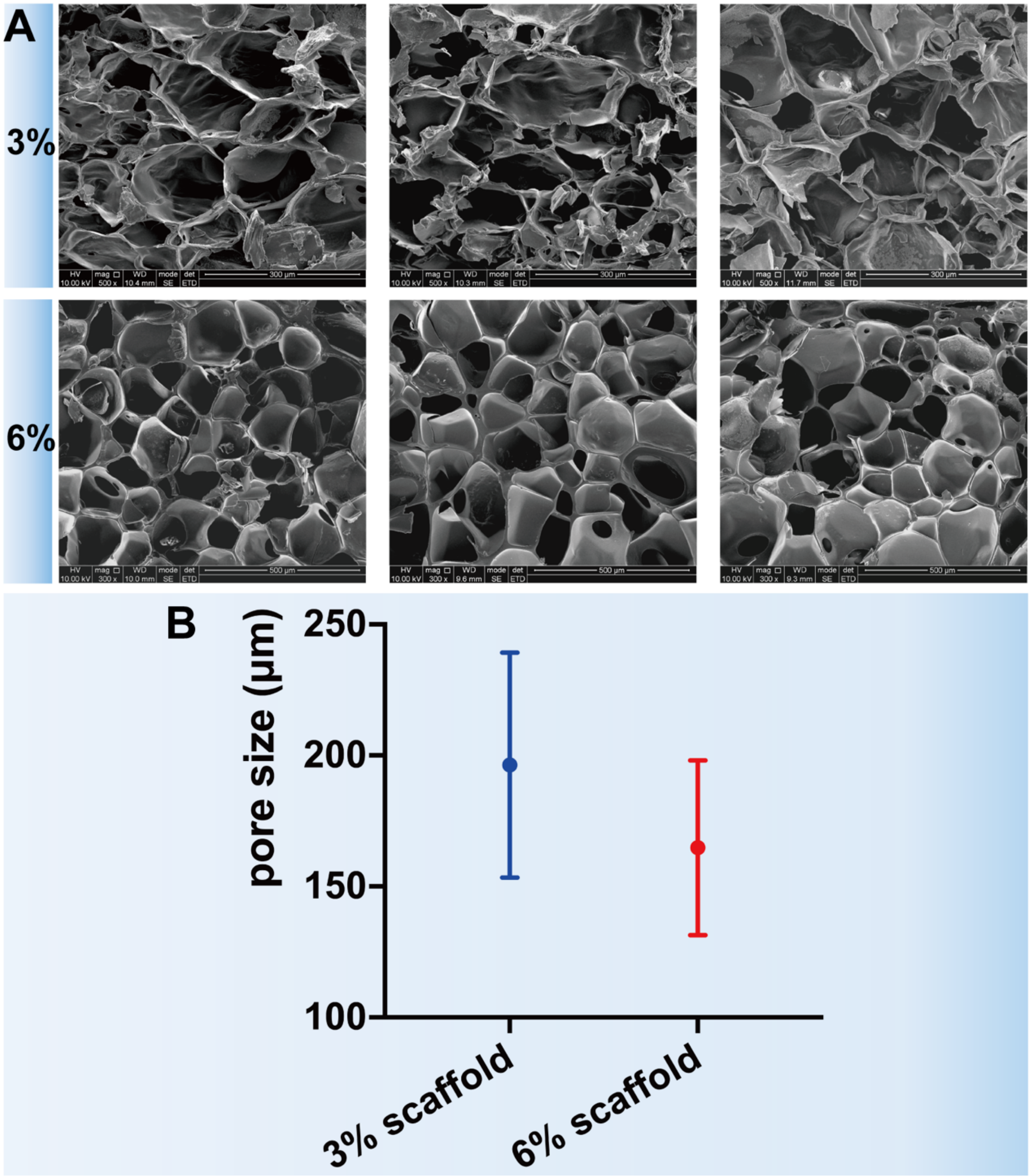

***Figure S1. (A)*** SEM images of hydrogel scaffolds with GelMA concentrations of 3% w/v and 6% w/v. ***(B)*** The average pore diameter of hydrogel scaffolds with GelMA concentrations of 3% w/v and 6% w/v.

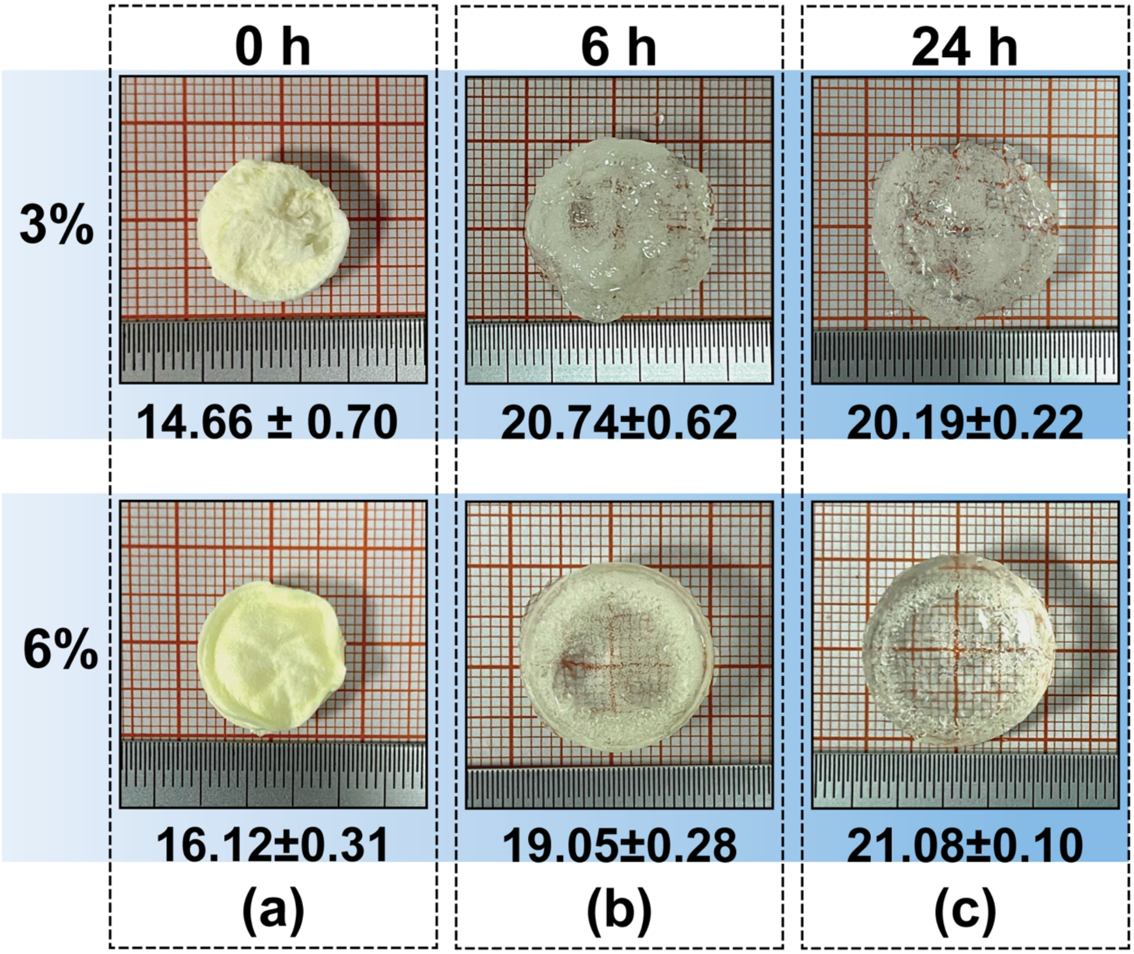


***Figure S2.*** The swelling properties of hydrogels with different GelMA contents in phosphate-buffered saline (PH = 7.4). The figure shows the diameter change of the soaked hydrogel (measured using ImageJ software). The experimental data are expressed as the mean ± standard deviation (mean ± SD).

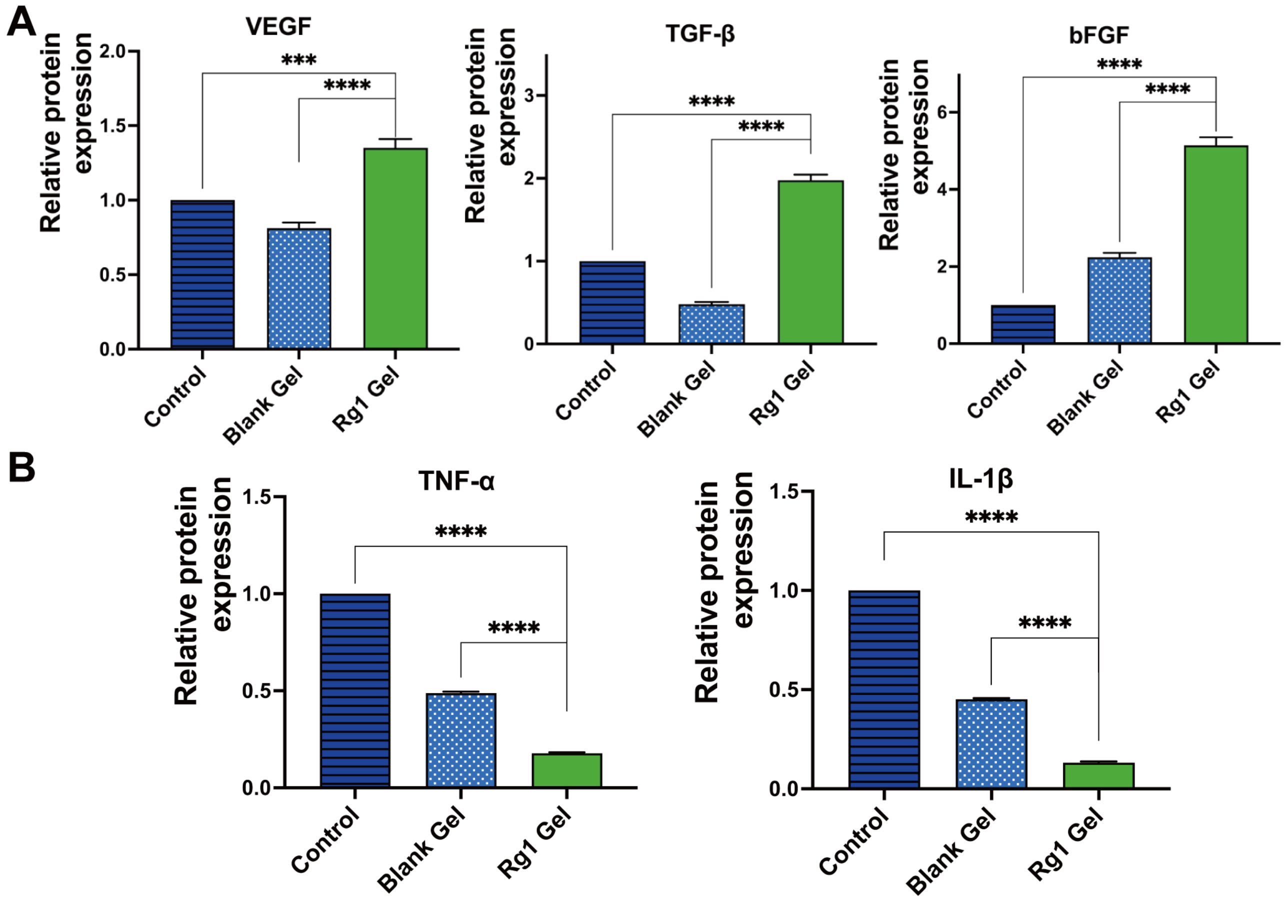


***Figure S3. (A).*** Cytokine protein expression analysis associated with soft tissue repair. ***(B).*** Inflammation-associated cytokines expression analysis. Differences were considered statistically significant at *$p < 0.05$, **$p < 0.01$, ***$p < 0.001$, ****$p < 0.0001$.
